# Supplementary material for: Rehabilitation interventions delivered via telehealth to support self-management of rheumatic and musculoskeletal diseases: A scoping review protocol
Source: PLoS One. 2024 Apr 16;19(4):e0301668. doi: 10.1371/journal.pone.0301668 (PMC11020871; doi:10.1371/journal.pone.0301668)
Supplement: S1 Appendix — (DOCX) [file pone.0301668.s001.docx]

**S1 Appendix. PRISMA-P checklist.**

**PRISMA-P (Preferred Reporting Items for Systematic review and Meta-Analysis Protocols) 2015 checklist: recommended items to address in a systematic review protocol***

| **Section and topic** | **Item No** | **Checklist item** | **Answer / destination within report** |
| --- | --- | --- | --- |
| **ADMINISTRATIVE INFORMATION** | | |  |
| Title: |  |  |  |
| Identification | 1a | Identify the report as a protocol of a systematic review | NA - report identified as a protocol for a scoping review. |
| Update | 1b | If the protocol is for an update of a previous systematic review, identify as such | NA |
| Registration | 2 | If registered, provide the name of the registry (such as PROSPERO) and registration number | Figshare - Available from: https://doi.org/10.6084/m9.figshare.19494179. See Methods Section. |
| Authors: |  |  |  |
| Contact | 3a | Provide name, institutional affiliation, e-mail address of all protocol authors; provide physical mailing address of corresponding author | Completed on the Title page. |
| Contributions | 3b | Describe contributions of protocol authors and identify the guarantor of the review | Author contributions completed within ‘Author contributions’ section, as per the CRediT taxonomy. Guarantor identified on Title page. |
| Amendments | 4 | If the protocol represents an amendment of a previously completed or published protocol, identify as such and list changes; otherwise, state plan for documenting important protocol amendments | NA |
| Support: |  |  |  |
| Sources | 5a | Indicate sources of financial or other support for the review | Completed within ‘Financial disclosures’ section. |
| Sponsor | 5b | Provide name for the review funder and/or sponsor | Completed within ‘Financial disclosures’ section. |
| Role of sponsor or funder | 5c | Describe roles of funder(s), sponsor(s), and/or institution(s), if any, in developing the protocol | Completed within ‘Financial disclosures’ section. |
| **INTRODUCTION** | | |  |
| Rationale | 6 | Describe the rationale for the review in the context of what is already known | Information provided in the Introduction Section |
| Objectives | 7 | Provide an explicit statement of the question(s) the review will address with reference to participants, interventions, comparators, and outcomes (PICO) | Completed. Please see the final paragraph and ‘Aims and research questions of the scoping review’ in the Introduction Section. Further, see ‘Eligibility criteria’ and ‘Search strategy’ in the Methods Section. Information found here pertains to the Population, Concept, Context framework. |
| **METHODS** | | |  |
| Eligibility criteria | 8 | Specify the study characteristics (such as PICO, study design, setting, time frame) and report characteristics (such as years considered, language, publication status) to be used as criteria for eligibility for the review | Completed within the Methods Section. Please see ‘Eligibility criteria’, ‘Types of evidence sources’, and ‘Search strategy’. |
| Information sources | 9 | Describe all intended information sources (such as electronic databases, contact with study authors, trial registers or other grey literature sources) with planned dates of coverage | Completed within the Methods Section. Please see ‘Types of evidence sources’, ‘Search strategy’, ‘Table 1’, and ‘Updated search’. |
| Search strategy | 10 | Present draft of search strategy to be used for at least one electronic database, including planned limits, such that it could be repeated | Completed within the Methods Section. Please see ‘Table 1’ and ‘Search strategy’. |
| Study records: |  |  |  |
| Data management | 11a | Describe the mechanism(s) that will be used to manage records and data throughout the review | Completed. Please see Methods Section. |
| Selection process | 11b | State the process that will be used for selecting studies (such as two independent reviewers) through each phase of the review (that is, screening, eligibility and inclusion in meta-analysis) | Completed. Please see ‘Sources of evidence selection’, ‘Title/abstract screening’, ‘Full-text review’, and ‘Updated search’ within the Methods Section. |
| Data collection process | 11c | Describe planned method of extracting data from reports (such as piloting forms, done independently, in duplicate), any processes for obtaining and confirming data from investigators | Completed. Please see ‘Full-text review’ and ‘Data extraction’ within the Methods Section. Also see ‘S2 Appendix’. |
| Data items | 12 | List and define all variables for which data will be sought (such as PICO items, funding sources), any pre-planned data assumptions and simplifications | Completed. Please see references to the PCC framework and the ‘Summarizing and presenting the findings’ within the Methods Section. Also see ‘S2 Appendix’. |
| Outcomes and prioritization | 13 | List and define all outcomes for which data will be sought, including prioritization of main and additional outcomes, with rationale | Scoping reviews are broad and iterative, thus anticipated outcomes are described within ‘Summarizing and presenting the findings’ and ‘S2 Appendix’, but more outcomes may be identified. |
| Risk of bias in individual studies | 14 | Describe anticipated methods for assessing risk of bias of individual studies, including whether this will be done at the outcome or study level, or both; state how this information will be used in data synthesis | NA |
| Data synthesis | 15a | Describe criteria under which study data will be quantitatively synthesised | Completed. Please see ‘Summarizing and presenting the findings’ in the Methods Section (e.g., frequency counts of PCC components). |
|  | 15b | If data are appropriate for quantitative synthesis, describe planned summary measures, methods of handling data and methods of combining data from studies, including any planned exploration of consistency (such as I^2^, Kendall’s τ) | As above. Frequency counts of PCC components. Planned exploration of consistency – NA. |
|  | 15c | Describe any proposed additional analyses (such as sensitivity or subgroup analyses, meta-regression) | NA (however, scoping reviews are iterative, thus data may be summarized across a range of categories) |
|  | 15d | If quantitative synthesis is not appropriate, describe the type of summary planned | Completed. Please see ‘Summarizing and presenting the findings’ within the Methods Section. Narrative summary of PCC components planned. |
| Meta-bias(es) | 16 | Specify any planned assessment of meta-bias(es) (such as publication bias across studies, selective reporting within studies) | NA |
| Confidence in cumulative evidence | 17 | Describe how the strength of the body of evidence will be assessed (such as GRADE) | NA |

*** It is strongly recommended that this checklist be read in conjunction with the PRISMA-P Explanation and Elaboration (cite when available) for important clarification on the items. Amendments to a review protocol should be tracked and dated. The copyright for PRISMA-P (including checklist) is held by the PRISMA-P Group and is distributed under a Creative Commons Attribution Licence 4.0.**

*From: Shamseer L, Moher D, Clarke M, Ghersi D, Liberati A, Petticrew M, Shekelle P, Stewart L, PRISMA-P Group. Preferred reporting items for systematic review and meta-analysis protocols (PRISMA-P) 2015: elaboration and explanation. BMJ. 2015 Jan 2;349(jan02 1):g7647.*
