# Supplementary material for: Rehabilitation interventions delivered via telehealth to support self-management of rheumatic and musculoskeletal diseases: A scoping review protocol
Source: PLoS One. 2024 Apr 16;19(4):e0301668. doi: 10.1371/journal.pone.0301668 (PMC11020871; doi:10.1371/journal.pone.0301668)
Supplement: S2 Appendix — (DOCX) [file pone.0301668.s002.docx]

**S2 Appendix. Data charting table.**

| **Author(s)** |  |
| --- | --- |
| **Year** |  |
| **Prior feasibility and pilot testing** |  |
| **Type of study**  (RCT, observational, case control study, qualitative, mixed methods (quant + qual), feasibility, pilot) |  |
| **Aims/purpose** |  |
| **Location/ setting** |  |
| **DEMOGRAPHICS:** |  |
| **Mean age (SD)** |  |
| **Female:** # (%) |  |
| **Population:** Condition(s) |  |
| **Inclusion/ exclusion criteria** |  |
| **Mean duration of symptoms/ time since diagnosis** |  |
| **INTERVENTION DESCRIPTION** |  |
| **Stage of development** (e.g. pilot/ feasibility testing, deployed) |  |
| **Strategies of development** |  |
| **Has a published/ validated development framework been cited? (Y/N)** |  |
| **Consideration of motivational theory? Y/N** (If yes, please specify theory used) |  |
| **Co-design with patients? Y/N** |  |
| **Co-design with HCPs? Y/N** |  |
| **Co-design with others? Y/N** |  |
| **Intervention purpose:**  Details of why the telerehabilitation intervention is being used (COVID-19, comparison, distance, costs, pilot, etc) |  |
| **Details of intervention:** |  |
| Autonomous/ Group based |  |
| Type of telehealth intervention (technology used) |  |
| Blended |  |
| Telephone |  |
| Website/device |  |
| Physical resource (e.g. booklet) |  |
| Videoconference |  |
| Exergame |  |
| **Terminology used** (telehealth, telerehabilitation, telemedicine) |  |
| **Definition of terminology** used (e.g. telehealth, telerehabilitation, telemedicine, etc) |  |
| **Summary of intervention schedule** |  |
| **Length of intervention** |  |
| **HCP interaction details** |  |
| What type of HCP is involved in interacting with patients during the study? |  |
| Asynchronous or synchronous or both (for technology use) |  |
| What level of interaction? (e.g. pre-defined over several visits, sporadic, patient-initiated, clinician-initiated) |  |
| What is the purpose of communicating/ interacting? |  |
| What form of communication/ interaction is used? (e.g. platform for communication - emails, telephone, F2F) |  |
| How often and for how long it the communication/ interaction? |  |
| Is any training/education provided to the patients on using the technology? |  |
| Is any training/education provided to the HCP on using the technology? |  |
| **Aspect of rehabilitation targeted- ‘Concept’** |  |
| - Education |  |
| - Disease management |  |
| - Remote monitoring (specify with or without feedback) |  |
| - Joint decision-making |  |
| - Psychological support |  |
| - Physical activity/ stretching |  |
| - Behaviour change techniques |  |
| - Physiotherapy techniques guided or created by a physiotherapist |  |
| - Occupational health (work-related guidance |  |
| - Lifestyle advice and support |  |
| - Social support |  |
| - Goal setting |  |
| - Clinical action plans |  |
| - Intelligent platform |  |
| - Others |  |
| **‘Context’ of telerehabilitation**   - Outpatient |  |
| - Home-based |  |
| - Community-based |  |
| - Not specified |  |
| **Measures of Effectiveness:** |  |
| Self-management support measures, e.g. patient activation, self-efficacy |  |
| What objective outcomes are used (e.g. accelermoter data, sit and stand test, 6-minute walk test)? |  |
| What clinician reported outcome measures (ClinROs), diaries or other tools are used? |  |
| What patient reported outcome measures (PROMs) are used? |  |
| What is used to measure intervention usability/satisfaction with intervention? |  |
| What is used to measure intervention adherence? |  |
| At what time points are outcomes assessed? |  |
| **RESULTS** |  |
| Was the intervention effective? |  |
| Adherence/ usability? |  |
| Conclusions, clinical implications, future directions? |  |
| Limitations (of the intervention) |  |
| **Methodological strengths/ limitations:** |  |
| Strengths (of the study design) |  |
| Limitations (of the study design) |  |
