# Supplementary material for: Rehabilitation interventions delivered via telehealth to support self-management of rheumatic and musculoskeletal diseases: A scoping review protocol
Source: PLoS One. 2024 Apr 16;19(4):e0301668. doi: 10.1371/journal.pone.0301668 (PMC11020871; doi:10.1371/journal.pone.0301668)
Supplement: S3 Appendix — (DOCX) [file pone.0301668.s003.docx]

**S3 Appendix. Charting evidence of ‘Concept’ and ‘Context’ for included studies.**

|  | **Concept- Aspect of rehabilitation** | | | | | | | | | | | | | | **Context- setting of telehealth** | | | | |
| --- | --- | --- | --- | --- | --- | --- | --- | --- | --- | --- | --- | --- | --- | --- | --- | --- | --- | --- | --- |
| **References** | **Education** | **Disease management** | **Remote monitoring (specify with or without feedback)** | **Joint decision-making** | **Psychological support** | **Physical activity/ stretching** | **Physiotherapy techniques guided or created by a physiotherapist** | **Occupational health (work-related) guidance** | **Behaviour change techniques** | **Social support** | **Lifestyle advice and support** | **Goal setting** | **Clinical action plans** | **Intelligent platform** | **Outpatient** | | **Home-based** | **Community-based** | **Not specified** |
|  |  |  |  |  |  |  |  |  |  |  |  |  |  |  |  |  | | | |
|  |  |  |  |  |  |  |  |  |  |  |  |  |  |  |  |  | | | |
