# Supplementary material for: Rehabilitation interventions delivered via telehealth to support self-management of rheumatic and musculoskeletal diseases: A scoping review protocol
Source: PLoS One. 2024 Apr 16;19(4):e0301668. doi: 10.1371/journal.pone.0301668 (PMC11020871; doi:10.1371/journal.pone.0301668)
Supplement: Attachment — Submitted filename: PLOS ONE Response to Reviewers_23FEB2024_v1.0.docx [file pone.0301668.s004.docx]

PONE-D-23-09456

Rehabilitation interventions delivered via telehealth to support self-management of rheumatic and musculoskeletal diseases: A scoping review protocol

PLOS ONE

## Author Response to Reviewers

Reviewer #1: Rehabilitation interventions delivered via telehealth to support self-management of rheumatic and musculoskeletal diseases: A scoping review protocol

**Reviewer comment:** Thank you for the opportunity to review this manuscript. The authors have conducted: A scoping review protocol to identify interventions delivered via telehealth for rheumatic and musculoskeletal diseases. I find the manuscript interesting and within the scoop of PLOS ONE journal. Please find my comments below.
**Author response:** Thank you for taking the time to review our manuscript, and for your helpful comments below. We have responded to each one in detail and have revised the manuscript accordingly.

**Reviewer comment:** I found a study similar with your review protocol! Please clarify this issue. (10.1093/rheumatology/keac133.287- Protocol for a scoping review to identify self-management support interventions delivered via telehealth for rheumatic and musculoskeletal diseases. Nuzhat Shakaib, Rosemarie Barnett, Raj Sengupta, Simon Jones, Peter C Rouse)

**Author response:** Thank you – this is indeed an abstract version of the protocol, which was presented at BSR 2022. However, there is not enough detail provided in the abstract to ensure transparency of our methodology and to limit the occurrence of reporting bias (see section 11.2 in the JBI Manual for Evidence Synthesis). Thus, we have decided to also publish the full protocol as a manuscript in a peer-reviewed scientific journal.

**Reviewer comment:** Add registration number in method section.

**Author response:** Thank you for this suggestion. However, to our knowledge, Figshare does not provide a registration number for protocols in the same way as other registers, e.g., PROSPERO. Instead, a doi is provided to facilitate citation. This doi/reference has already been cited in the manuscript, on page 8, line 177. The decision to register our protocol with Figshare is in alignment with recommendations within the JBI Manual for Evidence Synthesis, section 11.2. Scoping review protocols are not currently eligible for registration on PROSPERO.

**Reviewer comment:** State plan for documenting important protocol amendments.
**Author response:** Thank you – added (page 8, lines 178-181).

**Reviewer comment:** I think it is better to add the PRISMA 2020 flow diagram for reporting screening result.

**Author response:** Thank you – this is indeed how we intend to present our results, see line 317 of the manuscript. Further wording has been added to clarify this/ avoid confusion.

**Reviewer comment:** What Quality appraisal tool will be used to evaluate the quality of the eligible studies.

**Author response:** A quality appraisal tool will not be used to evaluate the eligible studies, due to the broad, exploratory nature of this review and the large heterogeneity of evidence identified (in terms of study design, methodology and populations – including both quantitative and qualitative sources of evidence). As indicated in the JBI Manual for Evidence Synthesis Appendix 11.2 PRISMA ScR Extension Fillable Checklist, a critical appraisal of individual sources of evidence is not required for a scoping review. Therefore, based on our aims and research questions (and considering our limited resources/time) we believe that a full critical appraisal was not warranted in this instance. However, we would recommend that any follow-up systematic reviews, with more specific research questions regarding effectiveness of telerehabilitation interventions, indeed perform appropriate risk of bias assessment. This decision will be justified in the final manuscript of the results.
